# Cultural and Socioeconomic Determinants of Hip and Knee Arthroplasty in the Medically Underserved Rio Grande Valley Community

**DOI:** 10.7759/cureus.91285

**Published:** 2025-08-30

**Authors:** Blake C Martin, Juan C Lopez-Alvarenga, John M Gaddis

**Affiliations:** 1 School of Medicine, The University of Texas Rio Grande Valley, Edinburg, USA; 2 Popultation Health and Biostatistics, The University of Texas Rio Grande Valley, Edinburg, USA; 3 Medical Education, The University of Texas Rio Grande Valley School of Medicine, Edinburg, USA

**Keywords:** arthroplasty, hip arthroplasty, joint replacement, knee arthroplasty, underserved

## Abstract

Introduction: Joint arthroplasty is a common procedure that is increasing worldwide. In this study, we aimed to discover if there were differences in demographics and social factors associated with individuals undergoing total knee arthroplasty (TKA) and total hip arthroplasty (THA) in the Rio Grande Valley (RGV). We hypothesized that older individuals and those with higher body mass index (BMI) would have an increased risk of TKA.

Methods: We conducted a retrospective chart review using the University of Texas Rio Grande Valley UTHealth electronic database from January 1, 2018, to July 1, 2024. Individuals were selected using Current Procedural Terminology codes for any patients with TKA or THA. Various statistical analyses were performed to analyze the data.

Results: We analyzed 252 patients with THA and 528 with TKA. The mean age was 69.9 (standard deviation, SD = 9.7) years, with a mean BMI of 31.8 (SD = 6.25). TKA was associated with higher BMI and was 20% more common in Hispanics than non-Hispanic patients: odds ratio = 2 (95% confidence interval = 1.4-3.3; p* *= 0.004). Factors associated with TKA compared with THA were age greater than 55 years, increased BMI, the use of public insurance, and Hispanic individuals. The probability of having TKA increased with BMI.

Conclusion: This study demonstrated the influence of unique cultural and socioeconomic conditions in the RGV in the context of TKA and THA. The findings suggest the need for policy interventions and continued research to analyze new policies and strategies to cope with the disparities in this community and other medically underserved regions.

## Introduction

Total joint arthroplasty (TJA) is one of the most common procedures performed worldwide, and the volume of these operations is on the rise. Joint arthroplasty includes removing a damaged joint and replacing it with a prosthesis [1]. This may entail replacing part of the joint or the joint as a whole, greatly improving the quality of life of the individual. Hips and knees are the most commonly replaced joints due to the stress they endure from bearing the weight of the upper body. However, other joints, such as shoulders, elbows, fingers, and ankles, may also be replaced [1]. The major indications for any joint replacement are degenerative joint disease, inflammatory arthropathy, osteonecrosis, and complicated fractures [2]. Risk factors that may increase an individual's probability of requiring joint replacement include being overweight, genetics, activities involving jumping, bearing excess weight, such as weightlifting, or any other activity that puts excess stress on joints. Joint arthroplasty is often recommended when other less invasive treatments, such as braces, canes, physical therapy, medicines, exercise, and weight loss, are not effective in relieving pain and improving mobility [1].

In 2010, the prevalence of total hip arthroplasty (THA) and total knee arthroplasty (TKA) in the United States was 0.83% and 1.52%, respectively [3]. Prevalence was higher among women than among men and increased with age, reaching 5.26% for THA and 10.38% for TKA at 80 years. This corresponds to about 2.5 million individuals (1.4 million women and 1.1 million men) with THA and 4.7 million individuals (3.0 million women and 1.7 million men) with TKA in 2010. Trends also indicate a significant increase in prevalence over time, with a shift to younger ages [3]. Another study states that predicted total annual counts (95% prediction intervals) for THA in the United States by 2020, 2025, 2030, and 2040 are (in thousands) 498 (475-523), 652 (610-696), 850 (781-925), and 1,429 (1,265-1,615), respectively [4]. For primary TKA, predicted total annual counts for 2020, 2025, 2030, and 2040 are (in thousands) 1,065 (937-1,211), 1,272 (1,200-1,710), 1,921 (1,530-2,410), and 3,416 (2,459-4,745), respectively [4]. When compared to the 2014 United States National Inpatient Sample numbers, the percent increases in projected total annual United States use for primary THA and TKA in 2020, 2025, 2030, and 2040 were as follows: primary THA, by 34%, 75%, 129%, and 284%; and primary TKA, 56%, 110%, 182%, and 401%, respectively. Primary THA and TKA were projected to increase for both women and men, in all age groups [4].

The Rio Grande Valley (RGV) is an area with underserved communities embedded in unique cultural and socioeconomic conditions that may influence healthcare decisions, like the decision of elective hip and knee arthroplasties. This population has a high prevalence of chronic conditions, such as obesity and diabetes, that can induce joint degeneration [5]. This region is also impoverished and medically underserved, and has a large population of undocumented immigrants [6,7]. The machismo construct may further contribute to the health of this region, as it may deter Hispanic men from seeking medical care, as it may be perceived as being feminine [8]. The study of demographic and social factors can demonstrate disparities in access to surgical interventions. This research can guide policymakers and administrations in allocating resources more effectively. In this study, we aimed to discover if there were differences in the demographics and social factors associated with individuals undergoing TKA and THA in the RGV. We hypothesized that individuals who are older and have higher body mass index (BMI) would have an increased risk of TKA.

## Materials and methods

Participants and procedure

We conducted a retrospective chart review using the University of Texas Rio Grande Valley (UTRGV) UTHealth electronic database. We requested and obtained IRB approval for this study via the UTRGV Institutional Review Board. All patients from January 1, 2018, to July 1, 2024, were analyzed and separated into two groups. These groups include patients who had TKA and those who had THA. Patients must have been diagnosed or undergone a procedure at an institution associated with the UTRGV to be included in the study. The age, sex, BMI, county of residence, race/ethnicity, and marital status were collected and analyzed for all patients. Individuals with partial joint arthroplasty or revision arthroplasty were not included in the study. Individuals with unilateral TJA and bilateral TJA were included by selecting patients with the Current Procedural Terminology (CPT) codes for any patient with TKA or THA. These codes include 27130 for total hip replacement and 27447 for total knee replacement. Duplicates in the medical charts were removed. Individuals who had more than one of the included procedures were counted as such. For example, an individual who underwent a right TKA and later required a left TKA would be considered two separate events. Incomplete demographic variables, such as ethnicity and marital status, were listed as such in the results (refused, unknown).

Data analyses

We used descriptive statistics with a mean (standard deviation, SD) or frequency (proportion) by measuring the dimension of each variable. The zip codes described the distribution of patients attending the Orthopedic clinic. Inferential statistics were done with mean contrast with the Student's t-test adjusted by variances, and for the size of effects, we used Cohen's d (95% confidence intervals, CIs). The risk of TKA was computed, considering the THA as a reference. We used logistic regression to calculate the odds ratio (OR) (95% CI) and the adjusted probabilities. The models included independent variables such as sex, age, BMI, marital status, and insurance status. The presented values of the OR can be interchanged using the inverse (1/OR) if we want to highlight the THA or TKA. The analysis was performed with Stata/MP version 19.0 (StataCorp, College Station, TX).

## Results

We included a sample of 780 participants, 252 (32.3%) with THA and 528 (67.7%) with TKA. Of these patients, 3.6% (9/252) of the THA patients have had bilateral THA, while 33.9% (179/528) of the TKA patients have had bilateral TKA. This does not necessarily indicate simultaneous joint arthroplasty, as the surgeries may have been spread out over time. The mean age at the procedure was 69.9 (age range = 15-96, SD = 9.7) years, with a mean BMI of 31.8 (SD = 6.25). TKA was associated with higher BMI and was about 20% more common in Hispanics (N = 422/718, 58.8% of the population with identified ethnicity) than non-Hispanic (N = 296/718, 41.2% of the population with identified ethnicity) patients: OR = 2 (95% CI = 1.4-3.3) p = 0.004; the inverse 1/OR was described in Table 1.

**Table 1:** Descriptive statistics of main variables and size of effect associated with the procedure ^†^OR of women and THA ^‡^OR of Hispanics and THA ^⁑^OR of commercial insurance and THA *p* value of <0.05 was significant SD: standard deviation; BMI: body mass index; OR: odds ratio; THA: total hip arthroplasty

Variable	Hip replacement	Knee replacement	Size of effect	*p* value
Sex (F/M)	137/115	300/228	0.9 (0.7 to 1.2)^†^	0.519
Age when procedure, years, mean (SD)	69.1 (11.1)	69.6 (9.0)	0.04 (-0.1 to 0.2)	0.556
BMI, kg/m^2^, mean (SD)	30.4 (7.2)	32.6 (10.2)	0.23 (0.07 to 0.40)	0.004
Ethnicity, n (%)				
Hispanic	104 (41)	318 (60)	0.5 (0.3 to 0.7)^‡^	<0.001
Non-Hispanic	121 (48)	175 (33)	-	-
Refuse	27 (11)	35 (7)	-	-
Marital status, n (%)				
Single	25 (10)	45 (9)	-	0.824
Married/partner	148 (59)	307 (58)	-	-
Divorced/separated	15 (6)	27 (5)	-	-
Widowed	20 (8)	40 (8)	-	-
Unknown	44 (17)	109 (21)	-	-
Insurance type, n (%)				
Commercial	79 (31)	122 (23)	1.5 (1.1 to 2.1)^⁑^	0.014
Medicare/Medicaid	173 (69)	406 (77)	-	-

**Figure 1:**
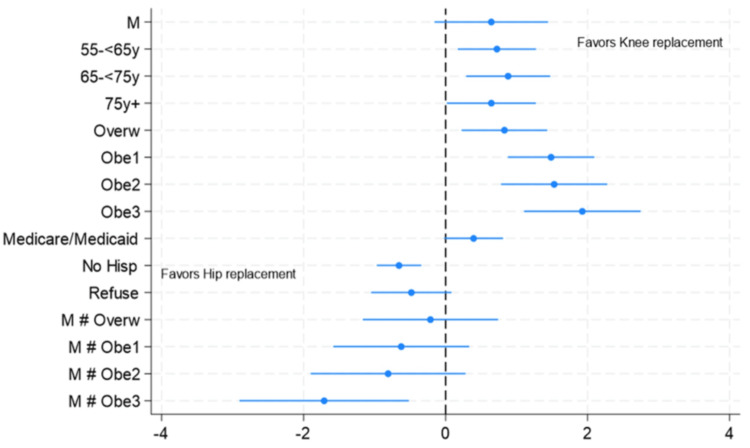
Risk of TKA compared with THA. The graph shows the Ln OR (95% CI) by all levels of the covariates. The reference groups were women, age <55, BMI <25, and commercial insurance TKA: total knee arthroplasty; THA: total hip arthroplasty; OR: odds ratio; CI: confidence interval; BMI: body mass index

Factors associated with TKA compared with THA were age greater than 55 years and increased BMI (Table 2). The use of public insurance was 1.5 (95% CI = 0.9-2.3) times more common for TKA. Non-Hispanic users were more likely to have THA: OR = 1.9 (95% CI = 1.4-2.6); p < 0.001. The risks are described in Table 2 and depicted in Figure 1.

**Table 2:** OR of TKA (THA as the reference) obtained from logistic regression. The only significant multiplicative interaction was sex and classes of BMI. Marital status did not play any role in the explanation or adjustments *p* value of <0.05 was significant OR: odds ratio; CI: confidence interval; BMI: body mass index; TKA: total knee arthroplasty; THA: total hip arthroplasty; BMI: body mass index

Variables	OR (95% CI)	*p* value
Women	Reference	-
Men	1.9 (0.85-4.24)	0.115
Age <55 years	Reference	-
55 to <65 years	2.06 (1.19-3.58)	0.01
65 to <75 years	2.41 (1.33-4.36)	0.004
More than 75 years	1.9 (1.02-3.56)	0.044
BMI <25 kg/m^2^	Reference	-
Overweight	2.29 (1.25-4.19)	0.007
Obe1	4.42 (2.4-8.12)	<0.001
Obe2	4.61 (2.18-9.75)	<0.001
Obe3	6.86 (3.02-15.59)	<0.001
Commercial insurance	Reference	-
Medicare/Medicaid	1.48 (0.98-2.25)	0.063
Hispanic	Reference	-
No Hispanic	0.52 (0.38-0.71)	<0.001
Refuse to answer ethnicity	0.62 (0.35-1.09)	0.094
Interaction F × BMI <25	Reference	-
Men × overweight	0.81 (0.31-2.09)	0.66
Men × obesity class 1	0.54 (0.21-1.39)	0.201
Men × obesity class 2	0.45 (0.15-1.32)	0.146
Men × obesity class3	0.18 (0.05-0.6)	0.005
Constant	0.34 (0.17-0.68)	0.002

**Figure 2:**
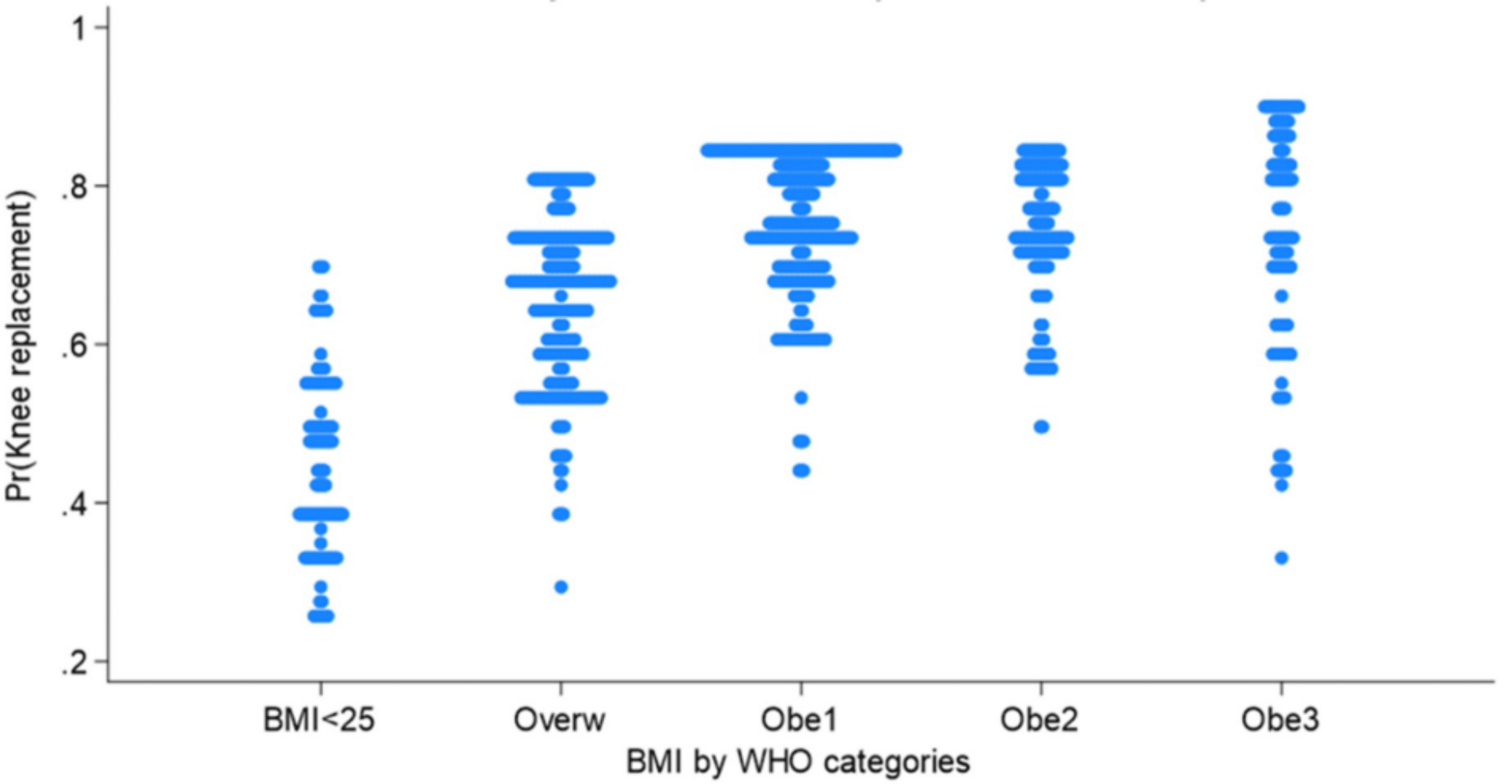
Predicted probability of having TKA compared with THA by BMI class adjusted by sex, age, ethnicity, and insurance TKA: total knee arthroplasty; THA: total hip arthroplasty; BMI: body mass index; WHO: World Health Organization; Overw: overweight

**Figure 3:**
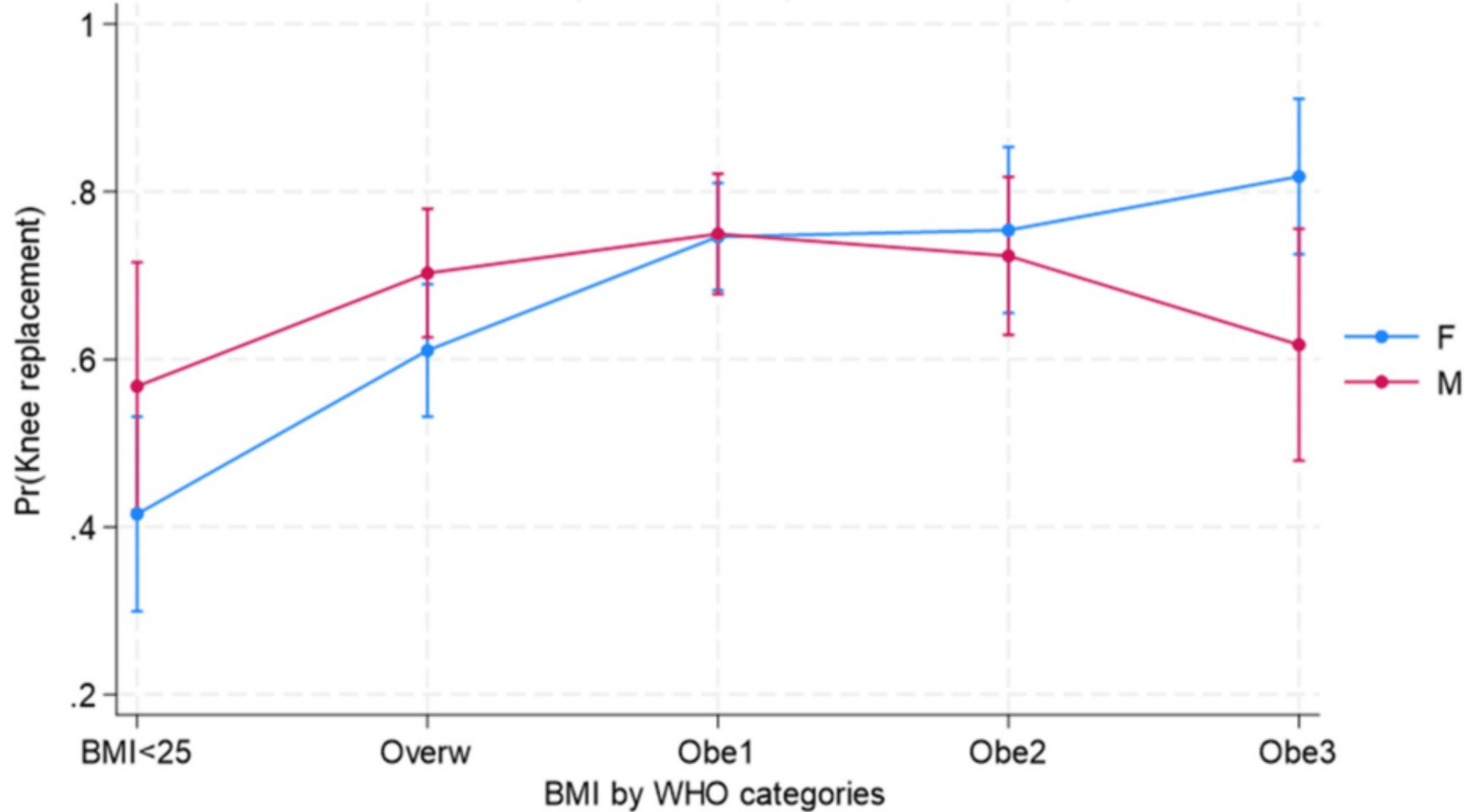
Predicted probabilities of TKA on the multiplicative interaction of sex and WHO categories of BMI TKA: total knee arthroplasty; BMI: body mass index; WHO: World Health Organization; Overw: overweight

The probability of having TKA increased with BMI (Figure 2), with a clear interaction of obesity class 3 in men (Figure 3).

## Discussion

The mean age of our patients who underwent a total joint (knee or hip) arthroplasty was 69.9 (SD = 9.7) years. This supports current literature as some previous studies have shown that the average age of knee arthroplasty is between 65 and 70 years [9-11]. Also, according to the American Academy of Orthopedic Surgeons, the average age of individuals who undergo knee or hip arthroplasty is between 50 and 80 years. The mean BMI of individuals in our study was 31.1 (SD = 6.25), which supports current literature, which states the average BMI for THA is around 29.1 and for TKA is around 32.4 [12,13]. Our study shows that TKA was associated with a higher BMI compared to THA, which also agrees with these studies [12,13]. This is because the mechanical load and biomechanical stresses affect the knee joint differently than the hip joint, accelerating degenerative changes in the knee joint [14,15]. The relationship between obesity and hip joint degeneration is less pronounced [14,15]. Previous literature has shown that Whites are more likely to have TJA than Blacks and Hispanics, which does not agree with the results in our study that indicate Hispanics are more likely to undergo TKA [16,17]. This could be because Hispanics made up the majority of our study population and the RGV region as a whole. This region is a poverty-stricken, medically underserved area with a large population of undocumented immigrants [6,7]. Also, the construct of machismo may contribute to this, as it may deter Hispanic men from seeking medical care because they perceive it as feminine [8]. Due to these factors, the individuals living in this region (predominantly Hispanics) may have poorer overall health, including joint health, leading to an increased frequency of TJA.

When comparing TKA and THA, our study found that age greater than 55 years old, increased BMI, use of public insurance, and being of Hispanic ethnicity were associated more with TKA. One study compared the average age of individuals undergoing THA and TKA and showed that the average age for THA is about 66 years, whereas the average age for TKA is 66.9 years [11]. Other studies show the average ages of TKA range from 65 to 70 years [9,10]. In contrast, THA can be seen to range from 65 to 65.4 years [18,19]. Collectively, these studies show that, on average, TKA occurs at a slightly later age than THA, which coincides with our study. Regarding BMI, our study results support current literature, which states that the average BMI for THA is around 29.1 and for TKA is around 32.4 [12,13]. This is due to the differences in load and stress mechanics between the two joints [14,15]. Regarding insurance, we did not find any studies in the current literature that compare the type of insurance and the utilization differences between THA and TKA. However, the literature does show that patients with Medicare, Medicaid, or those who self-pay have significantly lower odds of utilizing TKA compared to individuals with private insurance [20]. Another study shows that compared to commercial (private) insurance, individuals with Medicare, Medicaid, Self-pay, and workers' compensation had lower odds of THA [21]. Although there is no comparison, both THA and TKA are more likely to be utilized by individuals with private/commercial insurance. This may be due to the broader range of plans that private insurance offers, potentially including more comprehensive coverage. This may also be because these procedures are considered “elective,” and private insurance may offer quicker access to specialists and shorter wait times for elective procedures. Furthermore, private insurance plans may reimburse hospitals at a higher rate for arthroplasty procedures, as public insurance payout for these procedures has significantly declined in the past few decades [22].

Finally, to our knowledge, there have been no studies comparing the disparities in ethnicity between TKA and THA utilization. However, current literature has shown that individuals of certain minority groups (African Americans and Hispanics) have lower utilization of TKA and THA [23]. This is due to a combination of patient, provider, and system-level factors that collectively perpetuate disparities in access to joint arthroplasty [24-29]. Some of these factors include lower preference for surgery, higher levels of mistrust in the healthcare system, greater concerns for cost and recovery, and cultural beliefs that favor nonsurgical management. These attitudes persist after adjusting for socioeconomic status, access to care, and insurance, which demonstrates that the disparities are not explained only by financial barriers or disease burden [24-29].

This study has its limitations. Data were collected solely from UTRGV UTHealth electronic databases, so individuals who sought care at an institution not affiliated with UTRGV were not considered in this study. Therefore, this study may not be completely generalizable to the South Texas population. Most of the patients analyzed in this study were Hispanic, which indicates that our results may not be generalizable to the United States or another nation's general population whose ethnic landscape differs from the one in our study. The RGV is a poverty-stricken, medically underserved area with a large population of undocumented immigrants and individuals without health insurance [6,7]. This patient community is another reason the results of this study may not be universally applied. Furthermore, disease severity (radiographic OA grade) and comorbidities (e.g., diabetes) could impact results, so future studies should include these factors.

## Conclusions

High BMI and age were the major predictors of TKA compared with THA. Our study showed that as BMI increases and age is greater than 55, there is a higher probability of undergoing TKA. This association was evident in patients with obesity class 3, especially among men. Other factors were social disparities in the type of TJA. TKA was more common among Hispanic patients, while non-Hispanic patients were more likely to undergo THA.

This study demonstrates the influence of unique cultural and socioeconomic conditions in the RGV in the context of elective surgeries such as hip and knee arthroplasties. This finding suggests the need for policy interventions and continuing research to analyze new policies and strategies to cope with the disparities observed in this community as well as other underserved communities.

## References

[1] Nancy Garrick DD (2023). Joint replacement surgery: health information basics for you and your family. Published March.

[2] Taljanovic MS, Jones MD, Hunter TB, Benjamin JB, Ruth JT, Brown AW, Sheppard JE (2003). Joint arthroplasties and prostheses. Radiographics.

[3] Maradit Kremers H, Larson DR, Crowson CS (2015). Prevalence of total hip and knee replacement in the United States. J Bone Joint Surg Am.

[4] Singh JA, Yu S, Chen L, Cleveland JD (2019). Rates of total joint replacement in the United States: future projections to 2020-2040 using the national inpatient sample. J Rheumatol.

[5] (2023). Get out and go running: UTRGV’s South Texas Diabetes and Obesity Institute working to bring attention to diabetes. https://www.utrgv.edu/newsroom/2018/11/29-get-out-and-go-running-utrgv-s-south-texas-diabetes-and-obesity-institute-working-to-bring-attention-to-diabetes.htm.

[6] Kuruvilla R, Raghavan R (2014). Health care for undocumented immigrants in Texas: past, present, and future. Tex Med.

[7] Ramirez AG, Thompson IM, Vela L (2013). The South Texas Health Status Review. Springer Nature.

[8] Valdez LA, Jaeger EC, Garcia DO, Griffith DM (2023). Breaking down machismo: shifting definitions and embodiments of Latino manhood in middle-aged Latino men. Am J Mens Health.

[9] Ben-Shlomo Y, Blom A, Boulton C (2024). Outcomes after joint replacement 2003 to 2020. Outcomes after joint replacement.

[10] Price AJ, Alvand A, Troelsen A (2018). Knee replacement. Lancet.

[11] Springer BD, Levine BR, Golladay GJ (2021). Highlights of the 2020 American Joint Replacement Registry Annual Report. Arthroplast Today.

[12] Muthusamy N, Christensen T, Singh V, Sicat CS, Rozell JC, Schwarzkopf R, Lajam CM (2022). Trends of obesity rates between primary total hip arthroplasty patients and the general population from 2013 to 2020. Arthroplasty.

[13] Muthusamy N, Singh V, Sicat CS, Rozell JC, Lajam CM, Schwarzkopf R (2022). Trends of obesity rates between patients undergoing primary total knee arthroplasty and the general population from 2013 to 2020. J Bone Joint Surg Am.

[14] Derman PB, Fabricant PD, David G (2014). The role of overweight and obesity in relation to the more rapid growth of total knee arthroplasty volume compared with total hip arthroplasty volume. J Bone Joint Surg Am.

[15] Johnson CA, White CC, Kunkle BF, Eichinger JK, Friedman RJ (2021). Effects of the obesity epidemic on total hip and knee arthroplasty demographics. J Arthroplasty.

[16] Reyes AM, Katz JN (2021). Racial/ethnic and socioeconomic disparities in osteoarthritis management. Rheum Dis Clin North Am.

[17] Ibrahim SA (2007). Racial and ethnic disparities in hip and knee joint replacement: a review of research in the Veterans Affairs Health Care System. J Am Acad Orthop Surg.

[18] Patel I, Nham F, Zalikha AK, El-Othmani MM (2023). Epidemiology of total hip arthroplasty: demographics, comorbidities and outcomes. Arthroplasty.

[19] Ryan SP, Stambough JB, Huddleston JI 3rd, Levine BR (2024). Highlights of the 2023 American Joint Replacement Registry annual report. Arthroplast Today.

[20] Atarere J, Agudile E, Orhurhu V (2022). Racial and socioeconomic disparities in the utilization of TKA among patients with posttraumatic knee osteoarthritis: estimates from the United States national inpatient sample, 2011-2018. JB JS Open Access.

[21] Hartnett DA, Brodeur PG, Kosinski LR, Cruz AI Jr, Gil JA, Cohen EM (2022). Socioeconomic disparities in the utilization of total hip arthroplasty. J Arthroplasty.

[22] Catton E, Puddy A, Tyagi V, Kurkis GM, Shau DN (2024). The trend of Medicare reimbursement for total joint arthroplasty: using mathematical models to predict possible per-hour rate out to 2030. Arthroplast Today.

[23] Irgit K, Nelson CL (2011). Defining racial and ethnic disparities in THA and TKA. Clin Orthop Relat Res.

[24] Adu Y, Hurley J, Ring D (2024). Are there racial and ethnic variations in patient attitudes toward hip and knee arthroplasty for osteoarthritis? A systematic review. Clin Orthop Relat Res.

[25] Best MJ, McFarland EG, Thakkar SC, Srikumaran U (2021). Racial disparities in the use of surgical procedures in the US. JAMA Surg.

[26] Amen TB, Liimakka AP, Jain B, Rudisill SS, Bedair HS, Chen AF (2023). Total joint arthroplasty utilization after orthopaedic surgery referral: identifying disparities along the care pathway. J Arthroplasty.

[27] Okike K, Chang RN, Royse KE, Paxton EW, Navarro RA, Hinman AD (2022). Association between race/ethnicity and total joint arthroplasty utilization in a universally insured population. J Am Acad Orthop Surg.

[28] Katz JN, Arant KR, Loeser RF (2021). Diagnosis and treatment of hip and knee osteoarthritis: a review. JAMA.

[29] Burgesson B, Lethbridge L, Haase DA, Dunbar M (2025). Disparities in utilization rates of total knee and hip arthroplasty among racially visible populations in Canada: a retrospective cohort analysis. [Online ahead of print]. J Arthroplasty.

